# Clinical application of amplification-based versus amplification-free metagenomic next-generation sequencing test in infectious diseases

**DOI:** 10.3389/fcimb.2023.1138174

**Published:** 2023-11-29

**Authors:** Zhe-Ying Wang, Lu-Lu Li, Xue-Lei Cao, Ping Li, Jian Du, Ming-Jin Zou, Li-Li Wang

**Affiliations:** ^1^ Department of Clinical Laboratory, Qilu Hospital of Shandong University, Jinan, Shandong, China; ^2^ Shandong Engineering Research Center of Biomarker and Artificial Intelligence Application, Jinan, Shandong, China; ^3^ Department of Urology, The First Affiliated Hospital of Shandong First Medical University, Jinan, Shandong, China

**Keywords:** metagenomic next-generation sequencing, methodology, amplification-free method, PCR bias, infectious diseases

## Abstract

**Background:**

Recently, metagenomic next-generation sequencing (mNGS) has been used in the diagnosis of infectious diseases (IDs) as an emerging and powerful tool. However, whether the complicated methodological variation in mNGS detections makes a difference in their clinical performance is still unknown. Here we conducted a method study on the clinical application of mNGS tests in the DNA detection of IDs.

**Methods:**

We analyzed the effect of several potential factors in the whole process of mNGS for DNA detection on microorganism identification in 98 samples of suspected ID patients by amplification-based mNGS. The amplification-based and amplification-free mNGS tests were successfully performed in 41 samples. Then we compared the clinical application of the two mNGS methods in the DNA detection of IDs.

**Results:**

We found that a higher concentration of extracted nucleic acid was more conducive to detecting microorganisms. Other potential factors, such as read depth and proportion of human reads, might not be attributed to microorganism identification. The concordance rate of amplification-based and amplification-free mNGS results was 80.5% (33/41) in the patients with suspected IDs. Amplification-based mNGS showed approximately 16.7% higher sensitivity than amplification-free mNGS. However, 4 cases with causative pathogens only detected by amplification-based mNGS were finally proved false-positive. In addition, empirical antibiotic treatments were adjusted in 18 patients following mNGS testing with unexpected pathogens.

**Conclusions:**

Amplification-based and amplification-free mNGS tests showed their specific advantages and disadvantages in the diagnosis of IDs. The clinical application of mNGS still needs more exploration from a methodological perspective. With advanced technology and standardized procedure, mNGS will play a promising role in the diagnosis of IDs and help guide the use of antibiotics.

## 1 Introduction

Infectious diseases (IDs) remain one of the leading causes of morbidity and mortality among all patient populations worldwide (Nii-Trebi, 2017). A wide array of pathogens causes IDs; however, most infectious syndromes present with indistinguishable clinical manifestations. Accurate etiological diagnosis of IDs is always complex and challenging in the clinic (Messacar et al., 2017; Sinha et al., 2018). Conventional clinical microbiological assays usually refer to culture, immunological diagnostic experiments, and special serology tests (such as 1,3-β-D-glucan and galactomannan tests). By contrast, nucleic acid tests of pathogenic microorganisms take advantage of higher sensitivity and specificity (Peker et al., 2018; Olech, 2022). Metagenomic next-generation sequencing (mNGS) is a novel molecular test for unbiased full-coverage pathogen identification, which enables the simultaneous detection of all potential microorganisms in one experiment (Gu et al., 2021; He et al., 2022). Compared with routine targeted molecular tests such as polymerase chain reaction (PCR) and loop-mediated isothermal amplification (LAMP), mNGS is more suitable for the detection of pathogens without prior suspicion and can even be applied for novel or rare microorganism discovery (Han et al., 2020). Therefore, the mNGS test can provide more diagnostic evidence, especially for patients with emerging severe diseases or uncommon types of infection. To date, mNGS has been applied in clinical diagnosis as a powerful supplement to routine tests in a variety of IDs, including infections of the lower respiratory tract, bloodstream, and central nervous system (Dong et al., 2022; Li et al., 2022; Tong et al., 2022).

Though mNGS may serve as a new diagnostic tool to overcome the shortcomings of conventional methods, the complicated methodological variation limits its widespread use in the clinic (Simner et al., 2018). The workflow of mNGS involves multiple processes, including sample collection, optional host cell depletion, nucleic acid extraction, library construction, unbiased sequencing, bioinformatics analysis, and report interpretation. Without standard procedures, the complex processes in the mNGS test are accompanied by many problems in the final result interpretation (Gu et al., 2019; Gaston and Sinner, 2022). There still needs to be adequate awareness of whether the differences in the processes of mNGS detections affect their clinical performance. This study explored the clinical application of amplification-based and amplification-free metagenomic sequencing in DNA detection of IDs. The major difference in the methodology of amplification-based and amplification-free mNGS lies in whether a step of PCR amplification is necessary for the library construction (Beagan et al., 2021; Hsieh et al., 2021). Therefore, higher library content could be easily obtained in amplification-based mNGS than in amplification-free mNGS to provide more extensive sequencing data. The signal amplification also enlarges the reads number of the causative pathogens with shallow content, which has a significant meaning in the clinical diagnosis of IDs. However, it also brings possible contamination from aerosols of amplification products and bias toward specific sequences of certain lengths and GC content (Huptas et al., 2016; Luan et al., 2021). In the present study, we used mNGS for DNA detection on 98 samples from patients with suspected IDs. Amplification-based and amplification-free mNGS tests were compared in 43 of these samples to detect possible pathogens, sensitivity, and specificity for the clinical diagnosis of IDs. In addition, we analyzed the effect of several potential factors in the whole process of mNGS on microorganism identification for DNA detection.

## 2 Materials and methods

### 2.1 Sample collection and processing

This study was approved by the Qilu Hospital Ethics Committee and was performed following the Declaration of Helsinki. A total of 98 samples from 98 patients with suspected IDs were collected at Qilu Hospital of Shandong University (Jinan, Shandong Province, China) between August 2021 and April 2022. The clinical samples included peripheral blood, sputum, urine, abscess, joint, pleural, peritoneal, cerebrospinal, and bronchoalveolar lavage fluid (BALF). Peripheral blood samples were collected in EDTA or BCT tubes (BD) and centrifuged at 1,900 × g for 10 min to isolate plasma. Samples were stored at 4° and tested within 7 days of collection. Among them, 43 samples were detected using both amplification-based and amplification-free metagenomic sequencing.

### 2.2 Host cell depletion and DNA extraction

Host cell lysis was selectively performed on sanguinous or purulent samples. Briefly, the sample was incubated with saponin solution at a concentration of 0.025% at room temperature for 5 min, then adding 1 unit of Turbo DNase (Sigma) at 37 ° for 15 min. The DNase digestion was stopped according to the manufacturer’s instructions. Before DNA extraction, samples were pretreated at standard procedures according to their various types. One milliliter of plasma/cerebrospinal fluid (CSF) or 800 μl of other body fluid was pipetted into sterile tubes. All types of samples except for plasma were performed on bead bashing with shaking at 700 r.p.m for 30 min. Then DNA was extracted with a TIANamp Micro DNA Kit (Tiangen Biotech) per the manufacturer’s instruction. The extracted DNA was quantified using Qubit 3.0 fluorometer (Thermo Fisher).

### 2.3 Metagenomic next-generation sequencing

The DNA library for sequencing was prepared by enzymatic fragmentation (except for plasma-derived cell-free DNA), end repairing, terminal adenylation, and adaptor ligation using the NGS library construction kit (Enzymatics) according to the manufacturer’s protocol. For amplification-based library preparation, adapter-ligated DNA was subjected to PCR amplification with the following programs: initiation at 98 ° for 1 min, then 10 cycles of 98 ° for 20 s, 60 ° for 15 s, 72 ° for 30 s, and final extension of 72 ° for 5 min. For both methods, final DNA libraries were cleaned using Ampure beads (Beckman) and eluted in buffer EB (Qiagen). Qualified libraries were sequenced on Nextseq 550 sequencer (Illumina) per the manufacturer’s instruction. For each sequencing run, a negative control was included. The raw data was pre-processed using bcl2fastq2 software for the depletion of low-complexity and low-quality reads and trimming of adapters. Human sequence data were filtered according to GRCh38/hg38 by bowtie2 software. The remaining reads were aligned to a reference NCBI RefSeq database and in-house curated microbial genomic data using Burrows-Wheeler Aligner software to identify species, reads count, and relative abundance of microorganisms. Quality control filters: total reads > 10 million, GC ratio < 45%, Q20 > 85%, Q30 > 80% for each sample after sequencing. Following automatic pathogen detection, provisional reports were reviewed by a laboratory physician to interpret the results. In this study, the mNGS tests were laboratory-developed. Before being used in clinical testing, the performance of the mNGS tests was validated in accuracy, repeatability, and limit of detection. The performance validation was shown as follows: accuracy rate = 100%, coefficient of variation of reads number < 50%, limit of detection = 10^3^ copies/mL. The absolute values of correlation coefficients were higher than 0.9 in the detection of samples with serial dilution ratios. More details on the performance validation of the mNGS tests were shown in the supplemental file.

### 2.4 Other clinical or investigational tests for pathogen validation

Real-time PCR tests for *Aspergillus* and *Pneumocystis japonicum* were conducted using a multiplex PCR assay kit (XABT). PCR tests for *Pseudomonas aeruginosa* and *Escherichia coli* were conducted using CapitalBio multiplex PCR assay kit (CapitalBio Technology). PCR tests for *Mycobacterium tuberculosis*, Human betaherpesvirus 4, and Human betaherpesvirus 5 were conducted using corresponding PCR assay kits (DAAN GENE), respectively. The Xpert tests were performed using GeneXpert MTB/RIF detection system (Cepheid). Besides, conventional clinical assays in this study included culture, smear, tuberculous infection of T cell spot test (T-SPOT.TB), immunoassay, 1,3-β-D-glucan test (G test), and galactomannan test (GM test).

### 2.5 Statistical analyses

Statistical analyses were performed using the SPSS Version 23 software (IBM). Continuous data were compared using the Student’s t and Mann-Whitney tests. A comparison of paired continuous data was done using the McNemar test. Qualitative data were compared using the chi-square test. Calculations of sensitivity and specificity and their corresponding 95% CIs were performed by the Wilson-Brown test. A *p*-value < 0.05 was considered significant.

## 3 Results

### 3.1 Patient and sample characteristics

This study was conducted among 30 females and 68 males aged 21 to 92 years (mean = 58.7). Among the patients, 58 (59.2%) were immuno-compromised due to organ transplantation, chemotherapy, or drug-induced immunosuppression, and 83 (84.7%) were on antibiotics at the time of sample collection. A total of 98 samples were collected, including 25 peripheral blood, 19 BALF, 15 CSF, 12 sputum, 11 joint fluids, 7 peritoneal fluids, 4 abscesses, 3 pleural fluids, and 2 urine samples. All the samples were detected using amplification-based mNGS, while 43 of them were detected by amplification-free mNGS at the same time. The characteristics of the patients and samples are listed in **Table 1**.

**Table 1:** Patient and sample characteristics.

Patient characteristics	n = 98	n = 43
Age (years)		
Mean (Range)	58.7 (21~92)	57 (23~91)
Gender, n (%)		
Female	30 (30.6)	29 (67.4)
Male	68 (69.4)	14 (32.6)
Days hospitalized, median (Range)	14 (2~16)	13(0~60)
30-day mortality, n (%)	5 (5.1)	5 (11.6)
Immuno-compromised, n (%)	58 (59.2)	27 (79.4)
On empiric antibiotics at time of sample collection, n (%)	83 (84.7)	34 (79.1)
Primary diseases, n (%)		
Hematological diseases	20 (20.4)	15 (34.9)
Respiratory diseases	22 (19.4)	16 (37.2)
Neurological diseases	12 (12.2)	2 (4.7)
Gastrointestinal disease	11 (11.2)	2 (4.7)
Urological diseases	5 (8.2)	2 (4.7)
Cardiac disease	3 (3.1)	1 (2.3)
Endocrine disease	4 (4.1)	11 (2.3)
Other	10 (10.2)	4 (9.3)
Sample type, n (%)		
Peripheral blood	25 (25.5)	21 (48.8)
BALF	19 (19.4)	14 (32.6)
CSF	15 (15.3)	2 (4.6)
Sputum	12 (12.2)	3 (7.0)
Joint fluid	11 (11.2)	0
Peritoneal fluid	7 (7.1)	1 (2.3)
Abscess	4 (4.1)	1 (2.3)
Pleural fluid	3 (3.1)	1 (2.3)
Urine	2 (2.0)	0
Host cell depletion, n (%)	43 (43.9)	7 (16.3)

BALF, bronchoalveolar lavage fluid; CSF, cerebrospinal fluid.

### 3.2 Genus distribution of pathogen identification in mNGS and possible affected factors

**Figure 1:**
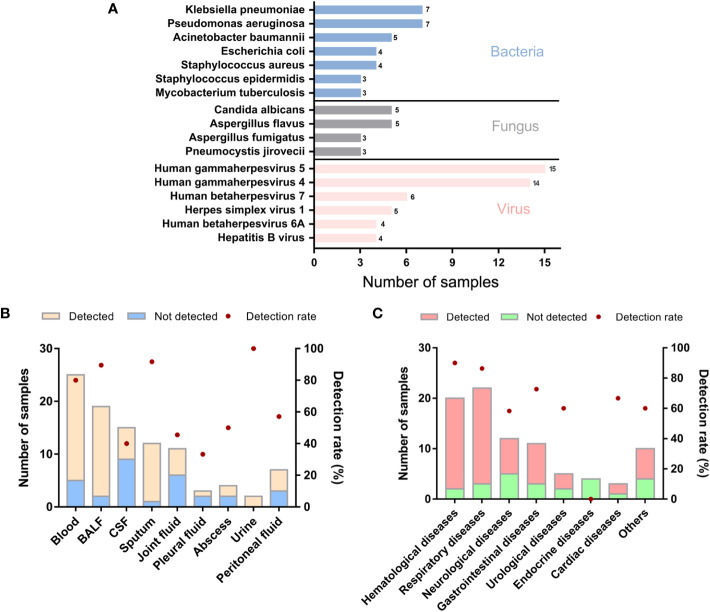
Genus distribution of mNGS results in 98 suspected ID patients. **(A)** Genus distribution of bacteria, fungi and virus detected by mNGS. **(B)** mNGS results of different sample types. **(C)** mNGS results of different primary diseases. BALF, bronchoalveolar lavage fluid; CSF, cerebrospinal fluid.

In the enrolled 98 samples, the detection rate of possible pathogens was 68.4% (67/98). Fifty-four pathogens were identified in total according to the amplification-based mNGS detection. Bacteria were the most commonly identified pathogens (n=34, 63.0%), followed by viruses (n = 12, 22.2%) and fungi (n = 8, 14.8%). No parasite was detected in these samples by the mNGS test. The most common bacteria, fungi, and viruses were *Klebsiella pneumoniae*, *Pseudomonas aeruginosa*, *Candida albicans*, *Aspergillus flavus*, and Human betaherpesvirus 5, respectively (**Figure 1A**).

We further analyzed whether potential factors in the whole process of mNGS for DNA detection could affect the microorganism identification in the 98 samples using amplification-based mNGS. The median concentration of the DNA extracted and libraries was 0.52 ng/μl (range 0.14 ~ 127 ng/μl) and 25.51 ng/μl (range 0.58 ~ 60.6 ng/μl), respectively. The mean read depth was 56.95 M (range 12.76 ~ 157.53 M). Of all the sample types (n > 5), the detection rate of possible pathogens was highest in sputum (11/12, 91.7%), then in BALF samples (17/19, 89.5%) (**Figure 1B**). Of all the primary diseases (n > 5), possible pathogens were more frequently detected in hematological (18/20, 90.0%) and respiratory diseases (19/22, 86.4%) (**Figure 1C**). We found that the detection of possible pathogens was significantly correlated with the concentration of DNA extracted (**Table 2**). It was more likely that a higher concentration of extracted nucleic acid was more conducive to the detection of microorganisms (*p* = 0.021). However, other potential factors, such as read depth, GC ratio, adaptor ratio, Q20, and Q30, had no significant differences in the groups of pathogen detected and not detected (*p* > 0.05). Therefore, under the corresponding quality control, these factors might not be attributed to the microorganisms’ identification in mNGS. In addition, there was also no significant correlation between the proportion of human reads, library concentration, the transformation efficiency of the library, amplification efficiency, and pathogen detection (*p* > 0.05) (**Table 2**).

**Table 2:** Possible factors affecting pathogen identification in mNGS.

Factors	Pathogen detected	Not detected	*p*-value
Sample number	67	31	–
Nucleic acid concentration (ng/μl), median (range)	0.66 (0.23~127)	0.37 (0.14~12.80)	0.021^*^
Library concentration (ng/μl), mean (range)	27.33 (0.79~57.80)	21.57 (0.58~60.60)	0.084
Transformation efficiency (%), median (range)	3936.08 (230.07~10181.80)	3464.29 (146.44~11000)	0.103
Amplification efficiency (%), median (range)	38 (6~120)	43 (1~157)	0.789
GC ratio (%), median (range)	42.80 (41.33~60.16)	42.88 (41.60~56.23)	1.000
Adaptor ratio (%), median (range)	20 (13~22)	20 (15~22)	0.347
Read depth, mean (range)	58.79 (127.61~157.53)	53.05 (18.98~100.40)	0.300
Q20 (%), median (range)	98.58 (94.64~98.84)	98.51 (96.53~98.99)	0.485
Q30 (%), median (range)	96.42 (90.31~98.09)	96.26 (90.09~97.38)	0.268
Non-human read number (M), median (range)	1.88 (0.28~23.33)	0.85 (0.21~31.38)	0.304
Proportion of human reads (%), median (range)	97.27 (22.33~99.11)	97.28 (26.88~99.23)	0.682

^*^p < 0.05.

### 3.3 Consistency of pathogen identified in amplification-based and amplification-free mNGS

**Figure 2:**
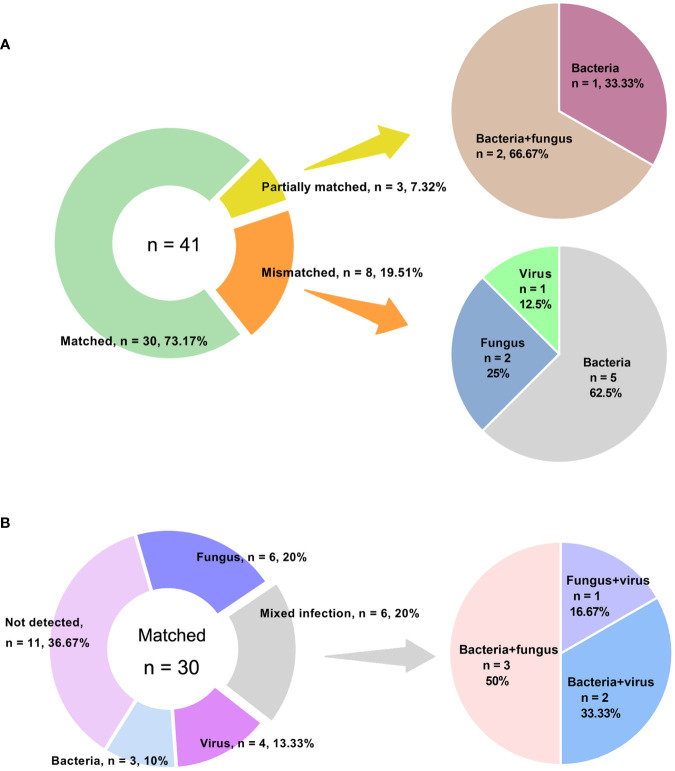
Concordance between amplification-based and amplification-free mNGS tests. **(A)** Samples were categorized as matched, partially matched and mismatched. Distribution of various infections in mismatched and partially matched groups were elucidated. **(B)** Detailed information of various infections in matched group.

Out of the 98 patients, we compared the performance of amplification-based mNGS to amplification-free mNGS in 43 cases (**Table 1**). Among them, 2 samples failed for amplification-free mNGS detection because of insufficient library yield. The median concentrations of libraries were 40.39 ng/μl (range 20.10 ~ 274.72 ng/μl) and 134.90 pg/μl (range 24.86 ~ 1012.93 pg/μl) in amplification-based and amplification-free mNGS, respectively. The read depth and number of non-human data in amplification-based mNGS were obviously higher than in amplification-free mNGS (*p* < 0.001). The two methods had no significant difference in Q30 (**Table 3**). In these cases, the detection rate of potential pathogens was 67.4% (29/43) in amplification-based mNGS and 51.2% (21/41) in amplification-free mNGS. The results of the two mNGS methods were completely matched in 30 (73.2%) cases, partially matched in 3 (7.3%) cases, and mismatched in 8 (19.5%) cases (**Figure 2A**). The agreement rates of bacteria, fungi, and virus identification were 62.5% (10/16), 80.0% (12/15), and 87.5% (7/8), respectively (**Figure 2B**). In the 8 discordant cases, amplification-free mNGS tests showed negative results, while amplification-based mNGS reported extra pathogens in all these cases. These microorganisms involved Hepatitis B virus, fungi such as *Pneumocystis jirovecii* and *Aspergillus flavus*, and bacteria such as *Stenotrophomonas maltophilia*, *Pseudomonas aeruginosa*, *Acinetobacter baumannii*, *Klebsiella pneumoniae*, *Staphylococcus epidermidis*, *Escherichia coli*, *Citrobacter braakii*, *Klebsiella aerogenes*, and *Enterobacter cloacae complex*.

**Table 3:** Possible affecting factors in the process of amplification-based and amplification-free mNGS.

Factors	amplification-based mNGS	amplification-free mNGS	*p*-value
Sample number	43	41	–
Library concentration (pg/μl), median (range)	40390 (20099~274720)	134.90 (24.86~1012.93)	< 0.001^***^
Read depth (M), mean (range)	65.53 (18.53~157.53)	34.67 (10.33~94.17)	< 0.001^***^
Number of non-human data (M), median (range)	1.46 (0.21~26.16)	0.31 (0.10~7.42)	< 0.001^***^
Q30 (%), median (range)	96.19 (88.82~97.03)	95.51 (93.97~97.20)	0.547

^***^p < 0.001.

### 3.4 Comparison of diagnostic performance between amplification-based and amplification-free mNGS

**Figure 3:**
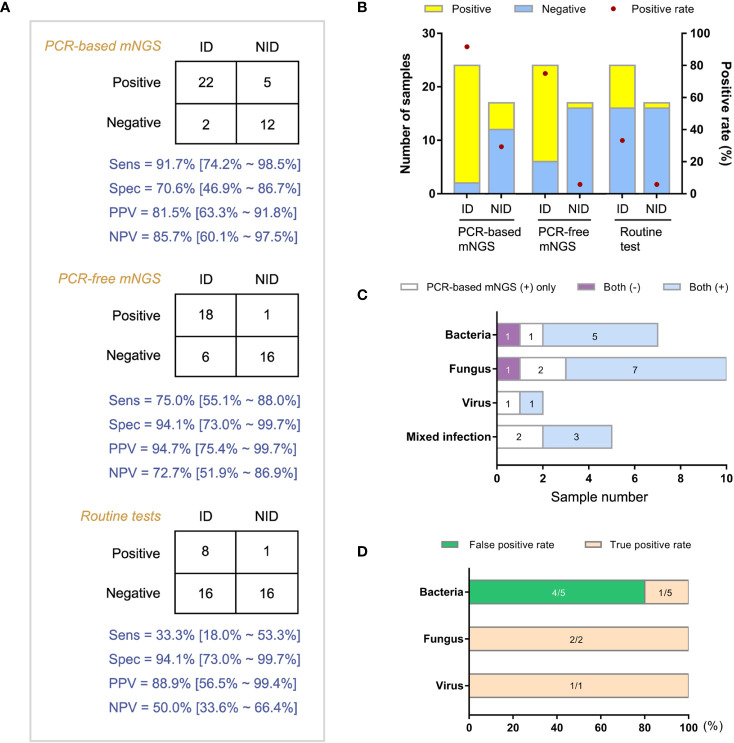
Comparison of diagnostic performance between amplification-based and amplification-free mNGS tests. **(A)** Modified 2×2 contingency tables according to the final clinical diagnosis. **(B)** The positive rates of mNGS and reference tests for ID and NID groups. **(C)** Distribution of various infections by two mNGS tests in ID group. **(D)** True positive rate and false positive rate of amplification-based mNGS in the mismatched group. ID, infectious disease; NID, non-infectious disease.

Forty-one patients with both amplification-based and amplification-free mNGS results were categorized into ID and non-ID (NID) groups according to the final clinical diagnoses with a retrospective and in-depth review. The overall agreement of mNGS tests in the diagnosis of ID was 78.0% (32/41). Then we compared the clinical performance of these two mNGS methods in the diagnosis of IDs (**Figures 3A, B**, n = 41). We found that the amplification-based mNGS test achieved approximately 16.7% higher sensitivity than amplification-free mNGS (91.7% vs 75.0%, *p* = 0.031). However, 4 cases with causative pathogens only detected by amplification-based mNGS were finally proved false-positive considering the results of routine culture/PCR tests, clinical characteristics, and therapeutic effects of the patients comprehensively. Even so, based on the results of this study, there was no significant difference in specificity or negative predictive value (NPV) between these two mNGS methods (**Figures 3C, D**). Compared with routine diagnostic tests (culture and PCR), both amplification-based and amplification-free mNGS significantly improved the detection rate of causative pathogens in ID patients (*p* = 0.001 and *p* = 0.006, respectively). In addition, the two mNGS methods presented similar specificity and NPV with routine diagnostic tests of IDs.

### 3.5 Clinical impact of amplification-based and amplification-free mNGS on antibiotic treatment

**Figure 4:**
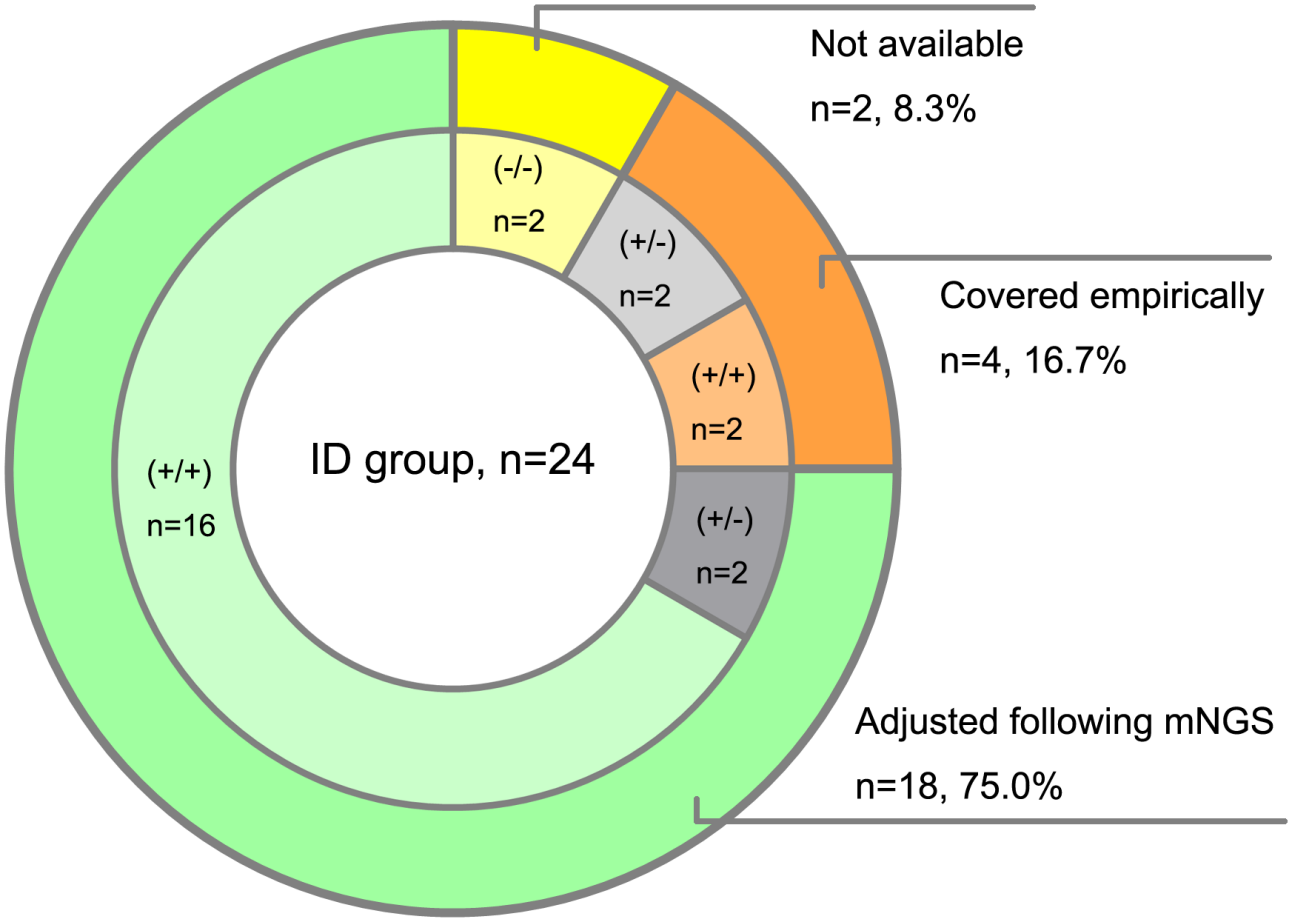
Clinical impact of mNGS tests on antibiotic treatment of ID patients. (+/+) represents both amplification-based and amplification-free mNGS positive, (+/-) represents only amplification-based mNGS positive, (-/-) represents both amplification-based and amplification-free mNGS negative.

Then we analyzed the true-positive samples to explore whether the antimicrobial drug regimens changed following mNGS tests in the clinic. Among the 24 ID patients detected by both mNGS methods, 8 cases were prompted for specific pathogens by conventional assays. Due to the shorter feedback time of mNGS than conventional methods, empirical antibiotic treatments were adjusted in 18 patients following the mNGS tests. Compared with the amplification-free mNGS test, 4 more ID patients could be detected with causative pathogens by amplification-based mNGS, 2 of which adjusted empirical antibiotic treatment (**Figure 4**).

## 4 Discussion

With recent technical development and lower costs, mNGS has become increasingly available for pathogen identification in the clinic. The main advantage of mNGS lies in its unbiased and hypothesis-free detection. mNGS test has been reported to achieve a high detection rate of causative pathogens in the diagnosis of IDs (Tao et al., 2022; Wei et al., 2022). The detection rate of suspected pathogens in mNGS varies from sample type and the study cohort (Han et al., 2019). Our results showed that 68.4% (67/98) of patients were detected with possible pathogens by mNGS. Of all the sample types (n > 5), the detection rate was highest in sputum (11/12, 91.7%), then in BALF (17/19, 89.5%) and blood samples (20/25, 80.0%). After interpreting the mNGS results considering the whole diagnosing and treating process, up to 40.8% (40/98) of patients were positive for causative pathogens in mNGS tests and diagnosed with IDs. The positive rate for causative pathogens was highest in BALF (15/19, 78.9%), then in sputum samples (9/12, 75.0%). However, the positive rate of blood samples decreased to 36.0% (9/25) (**Supplementary Figure 1**). The microorganisms detected in mNGS, which were finally interpreted as low or no pathogenicity, mainly included viruses with low load in blood and possible primary colonization bacteria from the upper respiratory tract. As a full-cover pan-pathogen detection method, the clinical interpretation of mNGS requires a comprehensive understanding of IDs and sufficient knowledge of microbiology.

Due to the complicated methodological variation in the processes of mNGS detection, the clinical application of mNGS is confronted with more challenges in the practical aspects (Mitchell and Simmer, 2019; Diao et al., 2022). Firstly, though with relatively high sensitivity, the mNGS test could not detect any pathogens in many ID patients (Qian et al., 2022). In previous clinical studies, the sensitivities of mNGS tests ranged from 66.7% to 90% (Han et al., 2020; He et al., 2022; Tao et al., 2022). The present study detected no plausibly causative pathogen in approximately 16 (16.3%, 16/98) patients that were finally diagnosed as IDs (Data not shown). Many interferences may lead to a false-negative result in the mNGS test. In the pre-analytic process, special sample characteristics such as hemolysis and jaundice, inappropriate sampling timing, and sample type are common causes of false negatives (Wang et al., 2020). Furthermore, here we explored whether potential factors in the whole analytic process of the mNGS test could affect the microorganism identification for DNA detection. Among the relevant parameters, we found that a higher concentration of DNA extracted was more conducive to detecting microorganisms. Sufficient content of nucleic acid could efficiently ensure the detection of causative pathogens, especially for pathogens with very low loads in the primary samples. Nevertheless, even with adequate nucleic acid input, the high proportion of human host DNA might mask the pathogen-derived sequences (Hasan et al., 2016; Marotz et al., 2018). However, we found no obvious correlation between the proportion of human reads and microorganism identification in this study. In addition, other parameters such as read depth or library concentration were not significantly attributed to the pathogen detection in mNGS. Actually, without standard procedures, the proposal design of mNGS tests varies from the beginning of nucleic acid extraction. Therefore, the interpretation of mNGS results in the clinic still needs more considerations from a methodological perspective.

Secondly, another major limitation of mNGS is the possibly higher rate of false positives in the diagnosis of IDs compared with routine methods (Huang et al., 2020). In previous clinical studies, the specificities of mNGS tests ranged from 59% to 81.4% (Han et al., 2020; He et al., 2022; Tao et al., 2022). In this study, the mNGS testing showed similar performance overall. In the mNGS test, potential exogenous contamination of microbial reads may derive from the reagents, consumables, environment, operations in the experiment, and strong positive samples in the same run (Bal et al., 2018; Zinter et al., 2019). Therefore, a template-free control is recommended to undergo all steps of the mNGS workflow in each run. In addition, signal amplification of specific pathogens in bioinformatic analysis could also bring risks of false positives (Breitwieser et al., 2019). Notably, the PCR amplification process in mNGS might be accompanied by the problems of aerosol contamination or bias towards specific sequences of certain lengths and GC content. In the present study, we compared the clinical application of amplification-based and amplification-free mNGS in IDs. We found that the two methods showed an agreement in 80.5% (33/41) of samples, including 14 negative and 19 positive cases. Of the 8 discordant cases, 4 cases of bacterial infections detected only by amplification-based mNGS were finally proved false positive. Conversely, the amplification-based mNGS test showed approximately 16.7% higher sensitivity (91.7% vs 75.0%) than amplification-free mNGS. The amplification-free mNGS test was unable to detect *Pneumocystis jirovecii*, *Aspergillus flavus*, and *Klebsiella aerogenes* in 3 blood samples, respectively, and Hepatitis B virus in 1 peritoneal fluid. It was likely due to the higher content of the library and more significant numbers of non-human data obtained in amplification-based mNGS, which provided much adequate microbial sequencing data for samples with low content of pathogen-derived DNA.

In this study, the amplification-based and amplification-free mNGS tests showed their specific advantages and disadvantages in differentiating ID and NID patients. Among the 24 true-positive samples clinically diagnosed as infectious diseases, eight were positive in culture or PCR. The concordance rate of the mNGS tests and culture/PCR was 100% in the eight samples, which showed that mNGS might exhibit high accuracy in detecting culture or PCR-positive samples. Furthermore, both methods could significantly improve the detection rate of causative pathogens compared with conventional diagnostic methods of IDs. There were also some limitations in this study. The patient number of the study cohort was relatively small, and the types of samples and diseases were diverse. In addition, the comparison of the two mNGS methods was also subject to more complicated factors than just the simple process of PCR amplification. In summary, the clinical application of mNGS still needs further exploration from a methodological perspective. So far, mNGS could not replace the current standard of routine diagnostic methods but should instead be used as an adjunct to these methods. mNGS could be considered when the standard of routine testing is unrevealing and can be used as a last resort effort to try to discern an infectious process. Alternatively, it may be considered for critically ill or severely immunocompromised patients where timely diagnosis is imperative for improved outcomes. At this point, further evidence is still required to establish its use in routine clinical care. With advanced technology and standardized procedure, mNGS will play a promising role in the diagnosis of IDs and, to a certain extent, help guide the use of antibiotics.

## Data availability statement

The data presented in the study are deposited in the Genome Sequence Archive repository, accession number CRA009951.

## Ethics statement

The studies involving humans were approved by the Qilu Hospital Ethics Committee. The studies were conducted in accordance with the local legislation and institutional requirements. The participants provided their written informed consent to participate in this study.

## Author contributions

Z-YW, M-JZ, and L-LW conceived the study. Z-YW, L-LL, X-LC, PL, and JD performed the experiments and analyzed the data. The first draft of the manuscript was writed by Z-YW, L-LL, and L-LW. All authors commented on previous versions of the manuscript. All authors had full access to all the data in the study and had final responsibility for the decision to submit for publication. All authors contributed to the article and approved the submitted version.
