# Supplementary material for: Clinical application of amplification-based versus amplification-free metagenomic next-generation sequencing test in infectious diseases
Source: Front Cell Infect Microbiol. 2023 Nov 29;13:1138174. doi: 10.3389/fcimb.2023.1138174 (PMC10716234; doi:10.3389/fcimb.2023.1138174)
Supplement: Supplementary file 1 [file DataSheet_1.docx]

Supplementary Material

Clinical application of PCR-based versus PCR-free metagenomic next-generation sequencing test in infectious diseases

Zhe-Ying Wang^1,2^, Lu-Lu Li^1^, Xue-Lei Cao^1,2^, Ping Li^1,2^, Jian Du^3^, Ming-Jin Zou^1^, Li-Li Wang^1,2,*^

*** Correspondence:** Li-Li Wang: liliwang@sdu.edu.cn

## Materials and Methods

## Performance validation of mNGS tests

## Before being used in clinical testing, the performance of the mNGS tests was firstly validated in accuracy, repeatability, and limit of detection. The clinical samples that the results of culture or PCR have verified were used to evaluate accuracy. The repeatability and limit of detection were evaluated using the biological reference materials. The reference materials of the microbes included Human betaherpesvirus 4 (China Center for Type Culture Collection, CCTCC GDV132), Staphylococcus aureus (Biobw, CMCC26003), Klebsiella pneumoniae (Biobw, CMCC46117), Streptococcus pneumoniae (Biobw, CMCC-B 31001), and Cryptococcus (BNCC, ATCC32719).

### The limit of detection validation of mNGS tests

## The above reference materials were mixed as a reference standard, which could cover microorganisms, including bacteria, fungi, and viruses (except parasites). The above microbes were diluted to the final concentration of 10^3^ copies/mL, respectively, to validate the limit of detection according to the relevant reference (Diao et al., 2022). Then the mNGS tests were repeatedly performed on the diluted reference standard for ten independent experiments. The criterion for validation is that the above microbes at 10^3^ copies/mL could be detected in all 22 repetitions.

### The accuracy validation of mNGS tests

## The clinical samples that the results of culture or PCR have verified were used to evaluate accuracy. The accuracy validation of mNGS tests was performed on ten clinical samples. The criterion for validation is that all the microbes proved by culture or PCR could be able to be detected in the mNGS testing.

### The repeatability validation of mNGS tests

## The mNGS tests were performed on the reference standard with serial dilution ratios (1×10^3^, 1×10^4^, and 1×10^5^ copies/mL) for three replications, respectively. Each corresponding microbe's coefficient of variation (CV) value was calculated using the sequence number normalized as in reads per million. The correlation coefficient of the concentration of the reference standard and the normalized sequence number was also calculated. Considering that the mNGS test was a qualitative experiment, the criterion for validation is that the CV value of each microbe could be less than 50%, and the absolute value of the correlation coefficient could be higher than 0.9.

## Reference

Diao, Z., Zhang, Y., Chen, Y., Han, Y., Chang, L., Ma, Y., et al. (2023). Assessing the Quality of Metagenomic Next-Generation Sequencing for Pathogen Detection in Lower Respiratory Infections. *Clin. Chem.* 69(9), 1038-1049. doi: org/10.1093/clinchem/hvad072

# Supplementary Figure

**
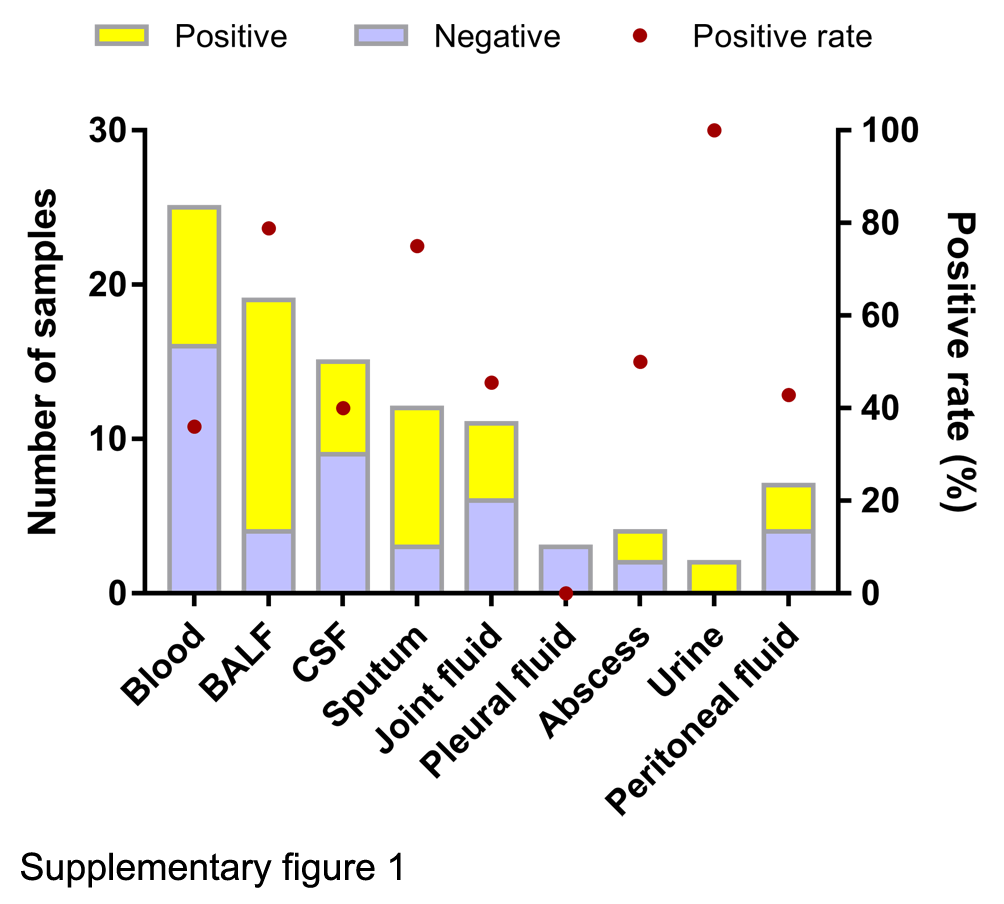
**

**Supplementary Figure 1.** mNGS results in different sample types according to final clinical diagnosis. BALF, bronchoalveolar lavage fluid; CSF, cerebrospinal fluid.
